# Structural basis for the substrate selectivity of PvuRts1I, a 5-hydroxymethylcytosine DNA restriction endonuclease

**DOI:** 10.1107/S139900471401606X

**Published:** 2014-08-29

**Authors:** Chen Shao, Chengliang Wang, Jianye Zang

**Affiliations:** a Hefei National Laboratory for Physical Sciences at Microscale and School of Life Sciences, University of Science and Technology of China, 96 Jinzhai Road, Hefei, Anhui 230026, People’s Republic of China; b Key Laboratory of Structural Biology, Chinese Academy of Sciences, Hefei, Anhui 230027, People’s Republic of China

**Keywords:** PvuRts1I, 5-hydroxymethylcytosine, endonuclease

## Abstract

5-Hydroxymethylation is a curious modification of cytosine that was discovered some decades ago, but its functional role in eukaryotes still awaits elucidation. 5-Hydroxymethyl­cytosine is an epigenetic marker that is crucial for multiple biological processes. The profile is altered under certain disease conditions such as cancer, Huntington’s disease and Alzheimer’s disease. Using the DNA-modification-dependent restriction endonuclease AbaSI coupled with sequencing (Aba-seq), the hydroxymethylome can be deciphered at the resolution of individual bases. The method is based on the enzymatic properties of AbaSI, a member of the PvuRts1I family of endonucleases. PvuRts1I is a modification-dependent endonuclease with high selectivity for 5-hydroxymethyl­cytosine over 5-methylcytosine and cytosine. In this study, the crystal structure of PvuRts1I was determined in order to understand and improve the substrate selectivity. A nuclease domain and an SRA-like domain are located at the N- and C-termini, respectively. Through comparison with other SRA-domain structures, the SRA-like domain was proposed to be the 5-hmC recognition module. Several mutants of PvuRts1I with enzymatic activity restricted to 5-hydroxymethylcytosine only were generated based on the structural analysis, and these enzyme variants are appropriate for separating the hydroxymethylome from the wider methylome.

## Abstract 2

The crystal structure of PvuRts1I was determined and a 5-hydroxymethylcytosine-binding pocket was identified in the SRA-like domain. Enzyme variants were engineered to assist in hydroxymethylome mapping based on the crystal structure of PvuRts1I.

## 1. Introduction   

5-Hydroxymethylcytosine (5-hmC) is the oxidized derivative of 5-methylcytosine (5-mC) and was first identified in *T*-even phages in the early 1950s (Wyatt & Cohen, 1952 ▶). After incorporation into genomic DNA, 5-hmC is normally glycosylated to protect phage DNA from digestion by bacterial restriction endonucleases during infection (Loenen & Raleigh, 2014 ▶; Kornberg *et al.*, 1961 ▶). During the 1970s, 5-hmC was shown to be present in the genomic DNA of several vertebrates, including rats, mice and frogs (Penn *et al.*, 1972 ▶; Penn, 1976 ▶). Recently, after the discovery that the ten-eleven translocation (Tet) family proteins are capable of converting 5-mC to 5-hmC, several reports demonstrated that 5-hmC is relatively abundant in mouse Purkinje neurons, granule cells and embryonic stem cells (Ito *et al.*, 2010 ▶; Kriaucionis & Heintz, 2009 ▶; Tahiliani *et al.*, 2009 ▶). A number of proteins were found to associate with 5-hmC using DNA pull-down experiments in combination with quantitative mass spectrometry, implicating that 5-hmC may be involved in multiple regulatory pathways *via* the recruitment of different reader proteins. In addition, the genomic distribution of 5-hmC is altered under several disease conditions, such as cancer, Huntington’s disease and Alzheimer’s disease (Bhattacharyya *et al.*, 2013 ▶; Bradley-Whitman & Lovell, 2013 ▶; Ficz *et al.*, 2011 ▶; Jin *et al.*, 2011 ▶; Pastor *et al.*, 2011 ▶; Stroud *et al.*, 2011 ▶; Wang *et al.*, 2013 ▶; Wu *et al.*, 2011 ▶).

Although cumulative evidence demonstrates that 5-hmC serves a critical role in various biological processes, full elucidation of its function has been hampered by the lack of a high-resolution distribution profile of 5-hmC in the genome. Consequently, extensive recent efforts have been made to develop new techniques to address this question. Currently, three methods that enable single-base resolution mapping of 5-hmC have been reported in the literature. The first, oxidative bisulfite sequencing (oxBS-seq), selectively converts 5-hmC to 5-formylcytosine (5-fC) using potassium perruthenate (KRuO_4_), which is subsequently read as thymine (Booth *et al.*, 2012 ▶, 2013 ▶). The second, Tet-assisted bisulfite sequencing (TAB-seq), uses TET1 to catalyze the conversion of 5-mC to 5-carboxycytosine (5-caC), whereas oxidation of existing 5-hmC is prevented by prior glucosylation. 5-hmC and 5-caC are read as cytosine and thymine, respectively (Yu *et al.*, 2012 ▶). Both methods combine selective oxidation with traditional bisulfite sequencing to distinguish between 5-mC and 5-hmC. The third method, AbaSI-coupled sequencing (Aba-seq), was designed to map the hydroxymethylome at single-nucleotide resolution in mammalian cells and is based on the high substrate selectivity of AbaSI, a member of the PvuRts1I family endonucleases, which have a high preference for 5-hmC over both 5-mC and cytosine (Sun *et al.*, 2013 ▶).

PvuRts1I family enzymes are classified as bacterial type IV modification-dependent restriction endonucleases and they are known to play an important role in defence against phage infection (Loenen & Raleigh, 2014 ▶). Several restriction endonucleases, such as McrBC, SauUSI and MspJI, have the ability to recognize and cleave double-stranded DNA containing modified cytosine residues including 5-mC and 5-hmC. However, they do not have the capacity to distinguish between 5-mC and 5-hmC owing to their structural similarity (Raleigh, 1992 ▶; Xu *et al.*, 2011 ▶; Zheng *et al.*, 2010 ▶). In contrast, most enzymes of the PvuRts1I family selectively bind to 5-hmC and 5-glucosylated methylcytosine (5-gmC) with high specificity over both 5-mC and cytosine, and cleave substrate DNA at a fixed distance from the modified cytosine (Borgaro & Zhu, 2013 ▶; Szwagierczak *et al.*, 2011 ▶; Wang *et al.*, 2011 ▶; Janosi *et al.*, 1994 ▶). The PvuRts1I family enzyme AbaSI was selected for use in Aba-seq to map the 5-hmC profile at high resolution (Sun *et al.*, 2013 ▶).

Several advantages of Aba-seq over the other two methods are apparent, including preservation of the DNA quality, higher efficiency in detecting 5-hmC at less abundant sites and the generation of semi-quantitative results (Sun *et al.*, 2013 ▶). However, technical hurdles still persist owing to the inherent enzymatic properties of AbaSI. Sun *et al.* (2013 ▶) claimed that AbaSI has no enzymatic activity towards 5-mC or cytosine, at least under the conditions studied (Sun *et al.*, 2013 ▶), and this may limit the application of this method. Moreover, Aba-seq requires the initial conversion of 5-hmC to 5-gmC (Sun *et al.*, 2013 ▶), which probably decreases the efficiency of the subsequent steps. Improvement of the substrate selectivity of PvuRts1I family enzymes towards 5-hmC, 5-mC and cytosine may overcome these problems.

To this end, we solved the crystal structure of PvuRts1I in this work and generated PvuRts1I enzyme variants based on structural analysis. The substrate selectivity of these mutants was evaluated and several possessed relatively higher enzymatic activities towards 5-hmC. These mutants could be used to decipher the hydroxymethylome and to separate it from the wider methylome in the future.

## 2. Materials and methods   

### 2.1. Cloning, expression and purification of PvuRts1I and mutants   

The sequence encoding PvuRts1I was synthesized using the optimized *Escherichia coli* codon set from Sangon Biotech (Shanghai) and then cloned into pET-28a vector (Novagen). *E. coli* Rosetta2 (DE3) cells (Novagen) carrying the vector were grown in LB medium at 310 K to an OD_600_ of 0.6–0.8 and were then induced with 0.4 m*M* isopropyl β-d-1-thiogalactopyranoside (IPTG) for 20 h at 289 K. Cells were harvested by centrifugation and lysates were prepared by sonication in lysis buffer (50 m*M* Na_2_HPO_4_, 300 m*M* NaCl, 5% glycerol pH 8.0), cleared by centrifugation and applied onto a nickel–nitrilotriacetic acid (Ni–NTA) Superflow column (Qiagen) pre-equilibrated with lysis buffer. Washing and elution were performed with lysis buffer containing 25 and 250 m*M* imidazole, respectively. The eluted proteins were applied onto a HiLoad 16/60 Superdex 200 gel-filtration column (GE Healthcare) in buffer *A* (20 m*M* Tris–HCl, 200 m*M* NaCl pH 8.0). Purified protein was concentrated to 6 mg ml^−1^ for crystallization. Mutations of PvuRts1I were generated by PCR using a pair of oligonucleotide primers designed with mismatching nucleotides at the target sites. Mutant proteins were expressed and purified under identical conditions to those used for wild-type PvuRts1I.

Selenomethionine-labelled PvuRts1I (Se-PvuRts1I) was expressed in *E. coli* Rosetta2 (DE3) cells in M9 medium supplemented with selenomethionine (Sigma–Aldrich) at a final concentration of 60 mg l^−1^ using the methionine-biosynthesis inhibition method. Se-PvuRts1I was purified identically to native PvuRts1I.

### 2.2. Crystallization, data collection, structure determination and refinement   

Crystals of Se-PvuRts1I were obtained using the sitting-drop vapour-diffusion method. 1 µl Se-PvuRts1I (6 mg ml^−1^) was mixed with 1 µl of a well solution consisting of 1.13 *M* NaH_2_PO_4_, 0.75 *M* K_2_HPO_4_, 0.1 *M* CAPS pH 10.5, 0.19 *M* Li_2_SO_4_, 6%(*v*/*v*) glycerol. The mixture was equilibrated against 100 µl well solution at 286 K. Single crystals were obtained after 4 d.

Crystals were soaked in a cryoprotectant solution consisting of well solution supplemented with 20%(*v*/*v*) glycerol and flash-cooled in liquid nitrogen. X-ray diffraction data were collected on beamline BL17U1 at the Shanghai Synchrotron Radiation Facility. Diffraction data for Se-PvuRts1I crystals were processed using *MOSFLM*, *POINTLESS* and *SCALA* from the *CCP*4 suite (Battye *et al.*, 2011 ▶; Evans, 2006 ▶; Winn *et al.*, 2011 ▶). The structure of Se-PvuRts1I was determined by the SAD method. The calculation of initial phases was performed using *AutoSol* and *AutoBuild* from the *PHENIX* software suite (Adams *et al.*, 2010 ▶) and the structure was determined by molecular replacement using *Phaser* (McCoy *et al.*, 2007 ▶). The structural model was manually refined to 2.9 Å resolution using *Coot* and *REFMAC*5 (Murshudov *et al.*, 2011 ▶; Emsley & Cowtan, 2004 ▶) with an *R* factor of 27.37% (*R*_free_ = 29.74%). The quality of the final model was validated using *PROCHECK* (Laskowski *et al.*, 1993 ▶). Data-collection and model-refinement statistics are shown in Table 1 ▶.

### 2.3. Preparation of DNA substrates   

To prepare the DNA substrates used in Figs. 2, 5 and 6 and Supplementary Fig. S4^1^, DNA fragments containing exclusively 5-hmC, 5-mC or unmodified cytosine were PCR-amplified from T4 genomic DNA by dATP/dGTP/dTTP mixed with dhmCTP using 5-hydroxymethyl-dCTP (Bioline), 5-methyl-dCTP (Fermentas) and dCTP, respectively. PCRs were carried out using KOD-Plus-Neo polymerase (Toyobo). The primers used were 5′-AGTTTTTGTATTGAAGT-3′ and 5′-TTAAA­TTAAATTAAAAAGGAAATAAAAATG-3′. These were the same as those used for the initial amplification (Wang *et al.*, 2011 ▶).

### 2.4. DNA restriction with PvuRts1I and mutants   

The PCR products were purified using the TIANquick Midi Purification Kit (Tiangen Biotech). For assessment of enzyme activity (Fig. 5), 10 µl substrate DNA (50 ng µl^−1^) was mixed with 1 µl PvuRts1I or the particular mutant (1 µg µl^−1^) and 2 µl NEB buffer 4 (50 m*M* potassium acetate, 20 m*M* Tris acetate, 10 m*M* magnesium acetate, 1 m*M* DTT pH 7.9) in a 20 µl final volume. The mixture was incubated for 1 h at 23°C and then resolved on a 1% agarose gel.

### 2.5. Relative selectivity of PvuRts1I and enzyme variants   

In each digestion series (Fig. 6), 100 ng substrate DNA was digested by PvuRts1I or an enzyme variant in a twofold serial dilution in NEB buffer 4. The protein concentration of the sample applied to lane 1 was 4 mg ml^−1^. The mixture was incubated for 1 h at 23°C and then resolved on a 1% agarose gel. The ratio of the relative selectivity was determined by comparison of the extent of digestion of different substrates.

## 3. Results   

### 3.1. Overall structure of PvuRts1I   

**Figure 1:**
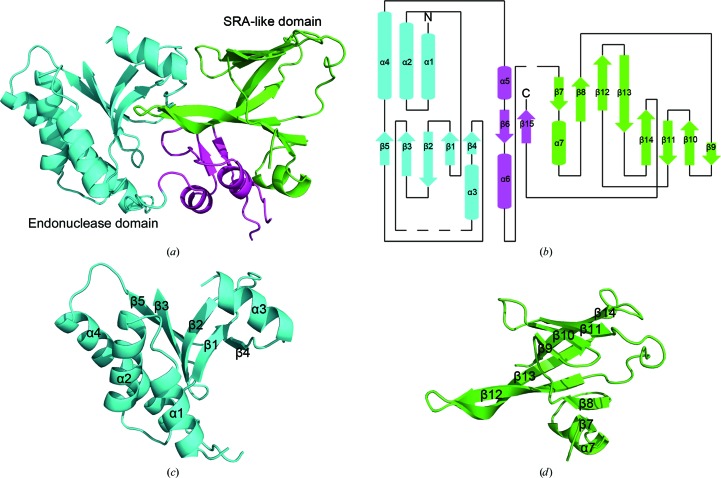
Overall structure of PvuRts1I. (*a*) Ribbon representation of PvuRts1I. The endonuclease domain and SRA-like domain are coloured cyan and green, respectively. (*b*) Schematic drawing of the topology of PvuRts1I. (*c*) Ribbon representation of the N-terminal endonuclease domain of PvuRts1I. (*d*) Ribbon representation of the C-terminal SRA-like domain of PvuRts1I.

To investigate the substrate specificity of the enzymes belonging to the PvuRts1I family, we solved the structure of full-length PvuRts1I. Although PvuRts1I was crystallized in the presence or absence of a 29 bp DNA fragment, we could only obtain crystals without substrate bound (Fig. 1 ▶ *a*). The PvuRts1I crystals diffracted to 2.9 Å resolution and phases were obtained by the single-wavelength anomalous dispersion (SAD) method. X-ray diffraction data-collection and structure-refinement statistics are shown in Table 1 ▶.

The structure of PvuRts1I consists of six α-helices and 15 β-strands, which fold into two distinct domains (Figs. 1 ▶ *a* and 1 ▶ *b*). The endonuclease domain is located in the N-terminal part and the DNA-binding domain constitutes the C-terminal portion (Figs. 1 ▶ *a* and 1 ▶ *b*). The two domains are connected by an irregular secondary structure which consists of a short α-helix (α5) and two β-strands packed against each other (β6 and β15). The N-terminal endonuclease domain adopts a typical three-layered α–β–α sandwich architecture, with a central five-stranded β-sheet flanked by three α-helices on one side and one α-helix on the other side (Figs. 1 ▶ *b* and 1 ▶ *c*). The C-terminal DNA-binding domain contains eight β-strands and one α-helix, giving an overall β-barrel-like structure with one side open. The concave surface of the open side houses the DNA-binding site (Figs. 1 ▶ *b* and 1 ▶ *d*).

**Figure 2:**
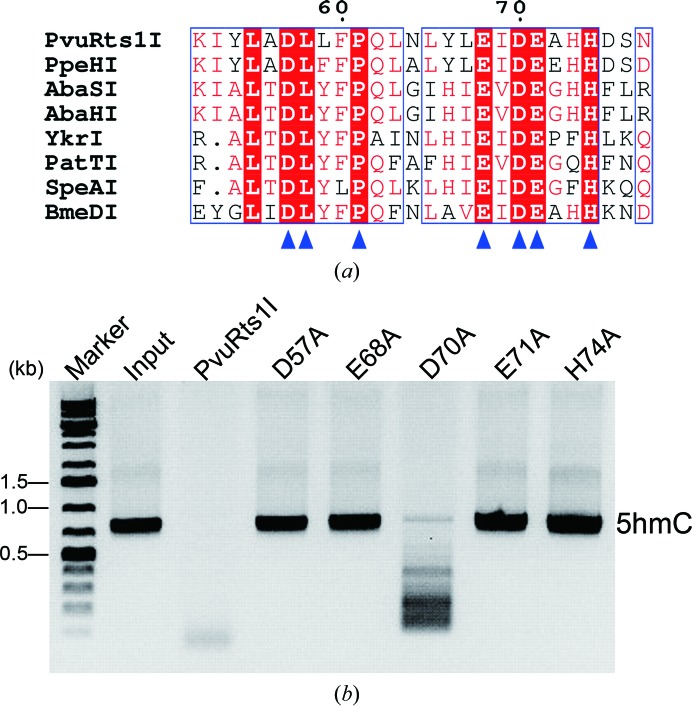
The conserved putative motif of the nuclease domain of PvuRts1I that is involved in metal-ion binding and catalysis. (*a*) Sequence alignment of the putative motif of PvuRts1I homologues. Absolutely conserved amino acids are indicated by blue triangles. (*b*) The *in vitro* modification-dependent enzymatic activity of PvuRts1I and its mutants on 5-hmC.

A search of the protein-structure database using *DALI* (Holm & Rosenström, 2010 ▶) revealed that the overall structure of the N-terminal domain is similar to the very short patch repair (Vsr) endonuclease, with a root-mean-square deviation (r.m.s.d.) of 2.4 Å (Tsutakawa *et al.*, 1999 ▶). This finding is consistent with previous reports that PvuRts1I belongs to the PD-(D/E)*X*K superfamily of endonucleases (Bujnicki & Rychlewski, 2001 ▶). However, part of the putative active site (amino-acid residues 71–76) was not visible in the electron-density map and could not be modelled, and may be highly flexible in the absence of the DNA substrate. A putative conserved motif proposed to be important for metal-ion chelation and enzymatic activity was previously identified in this domain (Fig. 2 ▶ *a*; Wang *et al.*, 2011 ▶). Asp57, Leu58, Pro61, Glu68, Asp70, Glu71 and His74 are absolutely conserved in PvuRts1I family enzymes. Accordingly, we generated several mutants and examined their enzymatic activity. As expected, the endonuclease activity of these mutants was abolished, except for the Asp70Ala variant, which retained some endonuclease activity (Fig. 2 ▶ *b*).

Comparison with structures in the PDB using the *DALI* server revealed that the C-terminal DNA-binding domain of PvuRts1I is related to 5-mC/5-hmC binding modules, including the SRA domains of *Arabidopsis* SUVH5 (*Z*-score = 4.9; r.m.s.d. = 3.1 Å) and human UHRF1 (*Z*-score = 4.4; r.m.s.d. = 3.1 Å) and the SRA-like domain of another modification-dependent restriction endonuclease MspJI (*Z*-score = 4.2; r.m.s.d. = 3.2 Å). Therefore, the DNA-binding domain of PvuRts1I is referred to as the SRA-like domain. As for other 5-mC/5-hmC binding modules, the overall shape of the SRA-like domain of PvuRts1I resembles a saddle, with a concave surface at the open side of the β-barrel. In other SRA or SRA-like domains the binding site of the modified cytosine is located on this concave surface. Similarly, we speculate that PvuRts1I recognizes 5-hmC using this surface.

### 3.2. Dimerization of PvuRts1I   

**Figure 3:**
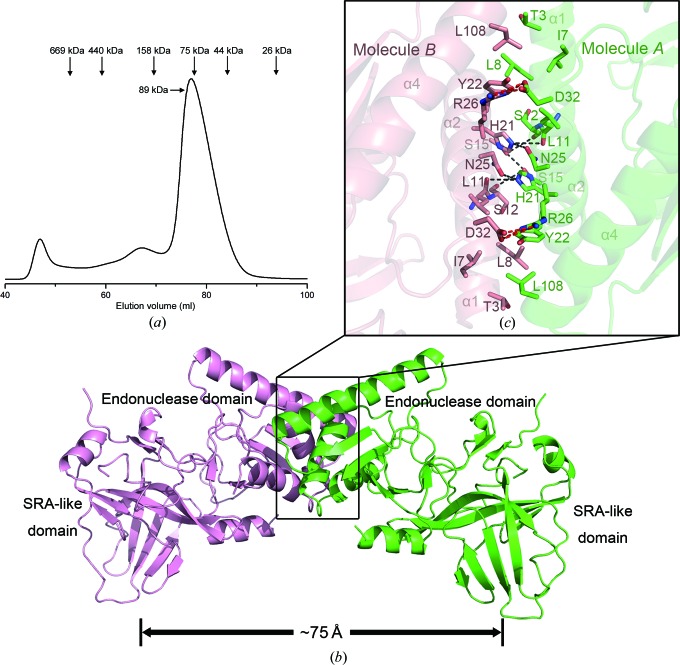
The dimeric assembly of PvuRts1I. (*a*) Size-exclusion chromatographic analysis of PvuRts1I. (*b*) Schematic drawing of the PvuRts1I dimer. The two subunits are coloured salmon and green, respectively. (*c*) An enlarged view of the dimer interface showing interactions between two neighbouring subunits.

Initial size-exclusion experiments indicated that PvuRts1I eluted as a single peak with an apparent molecular weight of 89 kDa; with a theoretical molecular weight of 34 kDa, this indicated the formation of a dimer in solution (Fig. 3 ▶ *a*), which is consistent with a previous report (Borgaro & Zhu, 2013 ▶). Only a single PvuRts1I molecule was present in the crystallographic asymmetric unit, and the dimer is constructed from a twofold symmetry-related molecule (Fig. 3 ▶ *b*). In addition, this dimer is also identified by the *PISA* server (Krissinel & Henrick, 2007 ▶). The interaction interface between the two molecules is formed by helices α1 and α2 of each subunit, and involves hydrophobic interactions and hydrogen bonds. Specifically, Ser15 and His21 of one subunit form hydrogen bonds to Leu11, Ser12, Ser15 and Asn25 of the opposite subunit and *vice versa*. In addition, Arg26 forms a salt bridge with Asp32, whilst Tyr22 and Leu108 contact Thr3, Ile6 and Leu6 *via* hydrophobic inter­actions (Fig. 3 ▶ *c*). The buried surface area of the interface is approximately 812 Å^2^, which is clearly adequate to stabilize the dimer in solution. This observation supports the idea that a dimer is the functional unit of PvuRts1I family endonucleases (Borgaro & Zhu, 2013 ▶).

### 3.3. The putative substrate-recognition site   

**Figure 4:**
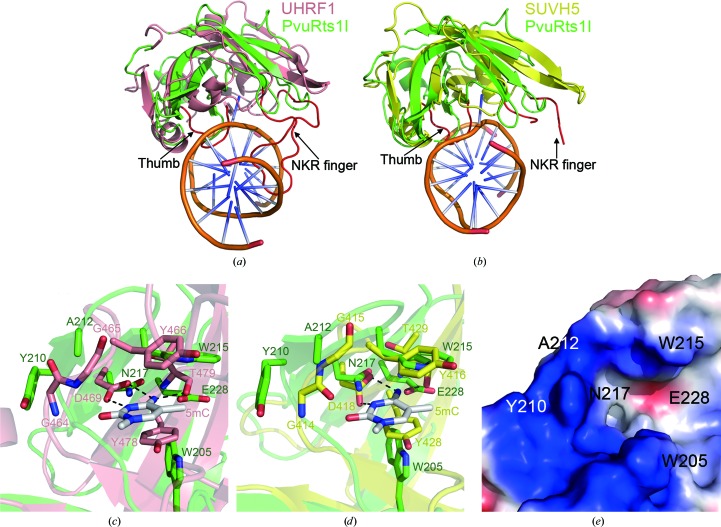
The putative substrate-recognition site of PvuRts1I. Comparison of the SRA-like domain structure of PvuRts1I (green) with the SRA-domain structure of (*a*) human UHRF1 (PDB entry 3clz, pink; Avvakumov *et al.*, 2008 ▶) and (*b*) *Arabidopsis* SUVH5 (PDB entry 3q0f, yellow; Rajakumara *et al.*, 2011 ▶) in complex with substrate DNA. The NKR finger and thumb loop are coloured red and indicated by arrows. A detailed view of the putative 5-hmC binding pocket superimposed on the 5-mC binding pocket of (*c*) human UHRF1 and (*d*) *Arabidopsis* SUVH5. The amino-acid residues and 5-mC are shown in stick representation. (*e*) Surface representation of the SRA-like domain of PvuRts1I. Amino-acid residues proposed to be involved in 5-hmC recognition are labelled.

As described above, the SRA-like domain of PvuRts1I is assumed to be the substrate-binding module. To shed light on the mechanism of substrate recognition, this domain was compared with those of other modified cytosine-binding modules (Qian *et al.*, 2008 ▶; Arita *et al.*, 2008 ▶; Avvakumov *et al.*, 2008 ▶; Hashimoto *et al.*, 2008 ▶; Rajakumara *et al.*, 2011 ▶). In SUVH5 and UHRF1, both the thumb loop and the NKR finger loop play important roles in 5-mC recognition (Figs. 4 ▶ *a* and 4 ▶ *b*). However, the NKR finger loop of PvuRts1I is much shorter than the equivalent loops of SUVH5 and UHRF1, suggesting that PvuRts1I might recognize the substrate DNA through the thumb loop.

In the SRA domains of SUVH5 and human UHRF1 in complex with DNA, the 5-mC base is flipped out of the DNA duplex and inserted into a binding pocket (Arita *et al.*, 2008 ▶; Avvakumov *et al.*, 2008 ▶; Hashimoto *et al.*, 2008 ▶; Rajakumara *et al.*, 2011 ▶). A similar but distinct pocket could be identified at the same position in the PvuRts1I SRA-like domain (Figs. 4 ▶ *c*, 4 ▶ *d* and 4 ▶ *e* and Supplementary Fig. S1). Although already narrow and deep in the case of ligand-free human UHRF1 (Supplementary Fig. S1*a*), this pocket becomes narrower and deeper still upon binding 5-mC owing to a quite dramatic conformational change (Supplementary Fig. S1*b*). At one end of the pocket there are two glycine residues that are potentially responsible for the conformational change owing to the intrinsic flexibility associated with this amino acid (Supplementary Fig. S1e). Similarly, the SUVH5 5-mC binding pocket is also narrow and deep (Supplementary Fig. S1*c*) and contains an equivalent pair of glycines (Supplementary Fig. S1*f*). In PvuRts1I, Tyr210 and Ala212 are found in place of these glycines, which are much more rigid than glycine (Supplementary Fig. S1*f*). As a result, the binding pocket of PvuRts1I may not be able to undergo this conformational change (Fig. 4 ▶ *e*). Sequence alignment shows that Tyr210 is conserved in the PvuRts1I homologues, indicating that it might contribute to substrate selectivity (Supplementary Fig. S2).

In both the human UHRF1 and SUVH5 SRA–DNA structures, the 5-mC base is sandwiched by π-stacking interactions between two aromatic amino acids: Typ466 and Tyr478 or Tyr416 and Tyr428, respectively (Figs. 4 ▶ *c* and 4 ▶ *d*). Interestingly, two tryptophan residues (Trp205 and Trp215) are located at equivalent positions in PvuRts1I, which provide aromatic stacking interactions with the 5-hmC of the substrate DNA (Figs. 4 ▶ *c* and 4 ▶ *d*). In addition, the side chains of Asp469 in UHRF1 and Asp418 in SUVH5 form hydrogen bonds to N3 and N4 of 5-mC, respectively. In PvuRts1I, Asn217 is situated at the same location, suggesting that this residue may perform a similar functional role (Figs. 4 ▶ *c* and 4 ▶ *d*). Furthermore, the main-chain carbonyl groups of Thr478 in human UHRF1 and Thr429 of SUVH5 make further hydrogen bonds to N4 of 5-mC. In PvuRts1I, Glu228 is positioned in this vicinity and may be involved in equivalent interactions (Figs. 4 ▶ *c* and 4 ▶ *d*). Sequence alignment of PvuRts1I homologues from different species indicates that the amino-acid residues proposed to interact with the modified cytosine are highly conserved (Supplementary Fig. S2) and therefore are likely to contribute to the substrate selectivity.

### 3.4. Improving the substrate selectivity of PvuRts1I   

**Figure 5:**
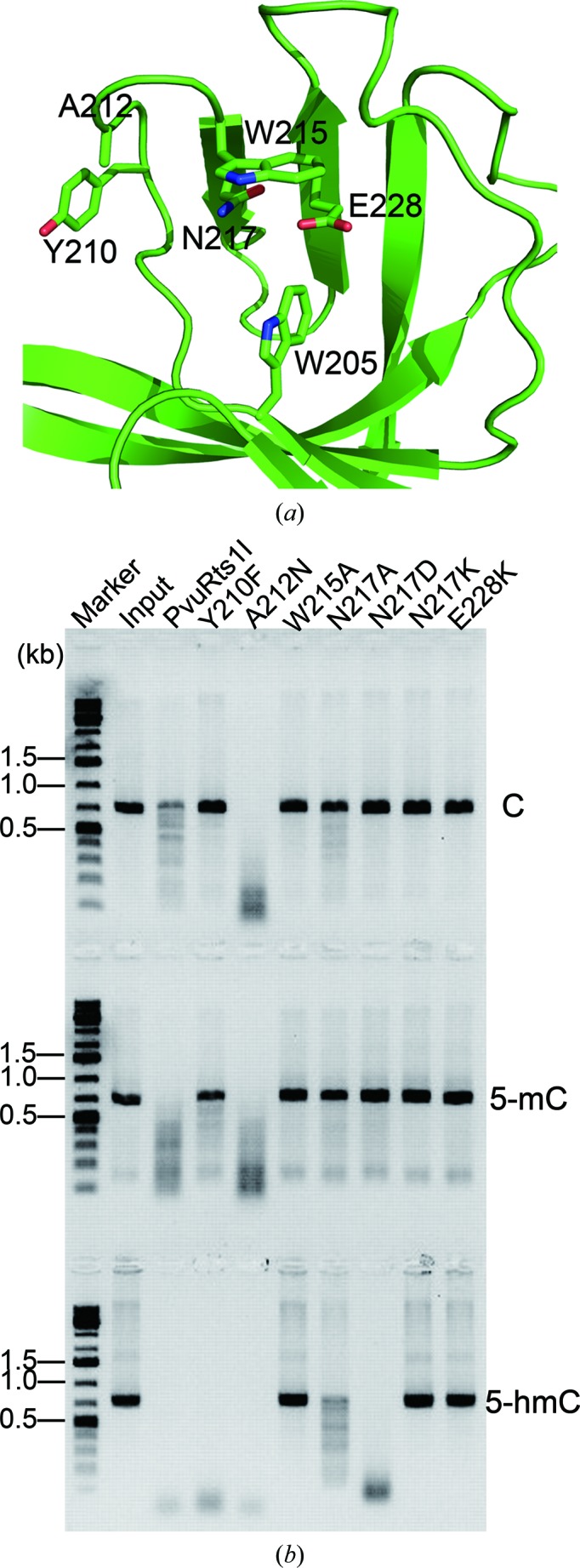
Improving the substrate selectivity of PvuRts1I *via* point mutations (see §[Sec sec2]2 for a description of the methods used). (*a*) Detailed view of the SRA-­like domain of PvuRts1I. Amino acids potentially involved in the recognition of 5-hmC are shown in stick mode. (*b*) Endonuclease activity of PvuRts1I and its mutants towards C (cytosine; upper panel), 5-mC (5-­methylcytosine; middle panel) and 5-hmC (5-hydroxymethylcytosine; bottom panel).

PvuRts1I was deemed appropriate for 5-hmC sequencing owing to its relative selectivity towards 5-hmC, 5-mC and cytosine, which is 2000:8:1, respectively (Szwagierczak *et al.*, 2011 ▶; Wang *et al.*, 2011 ▶). However, the residual activity of PvuRts1I towards 5-mC and cytosine decreases the accuracy of 5-hmC mapping in the genome owing to the lower abundance of 5-hmC relative to 5-mC and cytosine. Hence, improving the substrate selectivity of PvuRts1I would be beneficial for separating the hydroxymethylome from the methylome. Based on structural analysis, we engineered several point mutants of PvuRts1I (Y210F, A212N, W215A, N217A, N217D, N217K and E228K) and evaluated their substrate selectivity for 5-hmC, 5-mC and cytosine (Fig. 5 ▶).

Since wild-type PvuRts1I assembles into a functional dimer, we first examined the assembly of these mutants to confirm their correct oligomerization, which is probably required for activity. Fortunately, none of the mutations prevented the formation of a dimer in solution (Supplementary Fig. S3). The endonuclease activities of the W215A and E228K mutants were completely abolished (Fig. 5 ▶ *b*), presumably owing to the involvement of these residues in stabilizing the cytosine base in substrate DNA. Intriguingly, mutating Tyr210 to Phe and Ala212 to Asn exhibited opposite effects on substrate selectivity, despite their close proximity in the sequence. The preference of the the A212N mutant for 5-hmC over 5-mC and cytosine decreased significantly compared with the wild-type enzyme, while the selectivity of the Y210F variant increased (Fig. 5 ▶ *b*). Although the endonuclease activity of the N217K mutant was completely abrogated, the selectivity towards 5-hmC of the N217A mutant was enhanced (Fig. 5 ▶ *b*). Strikingly, the N217D mutant was found to retain similar endonuclease activity towards 5-hmC as wild-type PvuRts1I, while its activity towards 5-mC and cytosine was lost (Fig. 5 ▶ *b*).

**Figure 6:**
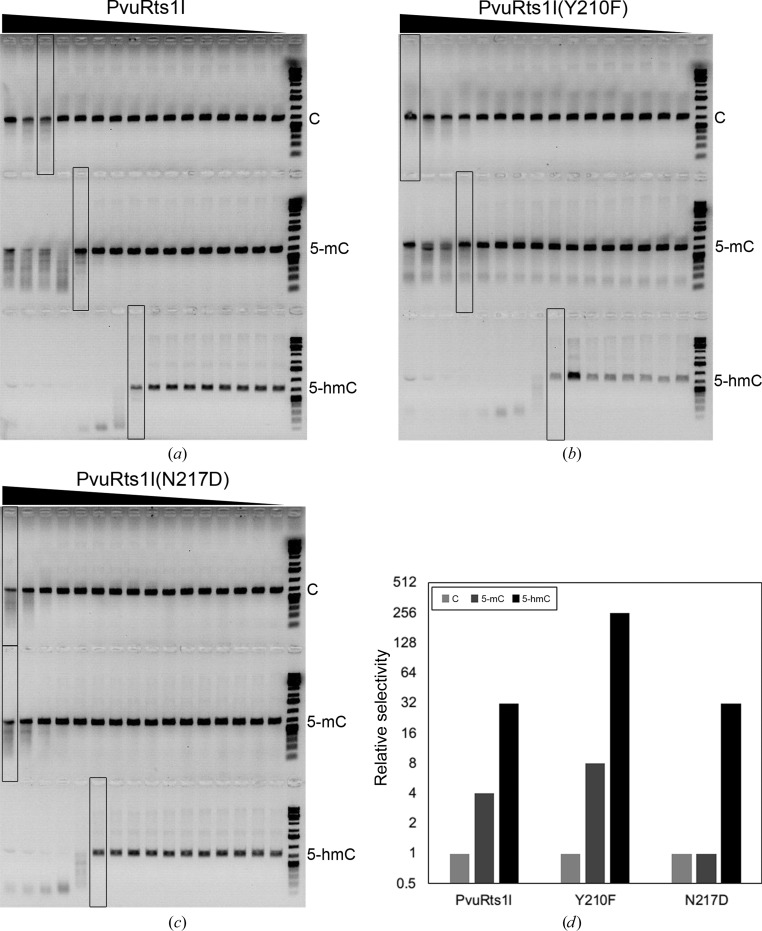
Relative selectivity of PvuRts1I and enzyme variants on unmodified cytosine (C), 5-mC and 5-hmC (see §[Sec sec2]2 for a description of the methods used). In each gel, the amount of enzyme is titrated from left (high) to right (low). All DNA substrates were made by PCR. (*a*) PvuRts1I; the approximate relative selectivity is 5-­hmC:5-mC:C = 32:4:1. (*b*) Y210F mutant; the approximate relative selectivity is 5-hmC:5-mC:C = 256:8:1. (*c*) N217D mutant; the approximate relative selectivity is 5-hmC:5-mC:C = 32:1:1. (*d*) Comparison of the relative selectivity towards 5-hmC, 5-mC and C for PvuRts1I and the Y210F and N217D mutants. The relative selectivity is plotted on a log scale and normalized based on the activity of C.

In order to further quantify the relative selectivity of PvuRts1I and its variants towards different cytosine modifications, we first investigated the appropriate reaction time for detecting relative selectivity. This showed that 5-hmC is digested in less than 10 min (Supplementary Fig. S4). However, in order to measure the relative selectivity of different modified cytosines, 60 min is a more appropriate reaction time. We then applied the same approach as previously used for PvuRts1I family enzymes (Wang *et al.*, 2011 ▶). Specifically, in each series 100 ng substrate DNA was digested by native PvuRts1I or a mutant in a twofold serial dilution. When the enzyme concentration was relatively high, PvuRts1I could digest DNA containing cytosine or 5-mC. We define the relative selectivity of PvuRts1I and its mutants as the ratio of specific activities on modified cytosines. On this basis, the relative selectivity of PvuRts1I is 5-hmC:5-mC:C = 32:4:1 (Fig. 6 ▶ *a*). For the Y210F mutant this was 256:8:ND (Fig. 6 ▶ *b*) and for the N217D mutant this was 32:1:1 (Fig. 6 ▶ *c*). Specificity towards 5-hmC was increased in the Y210F and N217D mutants (Fig. 6 ▶ *d*). The Y210F and N217D mutants are therefore probable candidates for distinguishing between 5-hmC and 5-mC.

## 4. Discussion   

In order to uncover the molecular mechanism underlying the relative selectivity towards 5-hmC, 5-mC and cytosine, we solved the crystal structure of PvuRts1I. The structure consists of two domains: a novel SRA-like domain that recognizes 5-hmC and an unusual nuclease domain that has not previously been curated by the NCBI Conserved Domain Database (Marchler-Bauer *et al.*, 2013 ▶). PvuRts1I homologues are widespread amongst bacterial species, and they share a similar substrate-sequence specificity despite relatively low amino-acid sequence identity (Borgaro & Zhu, 2013 ▶). However, these homologues may have evolved different substrate activities that may assist in survival in the wake of infection by T4-like phages (Borgaro & Zhu, 2013 ▶). During the course of our study, Kazrani and coworkers reported the crystal structure of PvuRts1I (Kazrani *et al.*, 2014 ▶). These two structure are very similar (with an r.m.s.d. of 0.4 Å for 247 aligned C^α^ atoms), apart from the N-terminal tag which was not cleaved in their work and two fragments (between residues 71 and 76 and between residues 181 and 184) which are disordered in our structure.

A recent report showed that endonucleases belonging to the PvuRts1I family form a functional dimer in solution (Borgaro & Zhu, 2013 ▶). Consistent with this, a dimeric assembly was observed both in solution and in the crystal structure of PvuRts1I (Fig. 3 ▶). Although Kazrani *et al.* (2014 ▶) claimed that the predicted dimer could not be found in their crystal, which belonged to space group *P*4_1_2_1_2, we considered that the dimer in our space group *P*4_3_2_1_2 crystal is a productive one. The PvuRts1I dimer interface is mediated by the N-terminal endonuclease domain, and the two SRA-like domains are located on opposite sides of the dimer (Fig. 3 ▶ *b*). The distance between the putative binding sites of the two SRA-like domains is 75 Å (Fig. 3 ▶ *b*). It has previously been shown that the recognition site of PvuRts1I is 5′-CN_11–13_↓N_9–10_G-3′/3′-GN_9–10_↓N_11–13_C-5′ (Szwagierczak *et al.*, 2011 ▶; Wang *et al.*, 2011 ▶). Two cytosines on opposite strands surrounding the cleavage site are necessary, and at least one cytosine should be modified for efficient cleavage (Wang *et al.*, 2011 ▶). The recognition sequence of PvuRts1I is approximately 22 bp long and 74 Å in length, which fits well with the distance between the two recognition sites in the dimeric PvuRts1I assembly.

In prokaryotes, there are two characterized proteins that can bind hydroxymethylated DNA, namely McrB and MspJI (Sukackaite *et al.*, 2012 ▶; Cohen-Karni *et al.*, 2011 ▶). However, neither of these is able to distinguish between 5-mC and 5-hmC. PvuRts1I harbours an SRA-like domain that is similar to MspJI, *Arabidopsis* SUVH5 and human UHRF1. There are some key differences that enable PvuRts1I to recognize 5-hmC specifically. The binding pocket of PvuRts1I is shallower and wider than that in SUVH5 and UHRF1 (Supplementary Figs. S1*a*–S1*d*). In addition, comparison of the UHRF1 SRA domain in the presence or absence of substrate DNA revealed a large conformational change in the 5-mC binding pocket after insertion of 5-mC. Two glycine residues on one edge of the binding pocket may be responsible for this induced-fit mechanism of 5-mC recognition. In PvuRts1I, Tyr210 and Ala212 replace these glycine residues, which results in a more rigid pocket of fixed shape and size (Supplementary Figs. S1*e* and S1*f*). The crystal structure of PvuRts1I bound to the 5-hmC DNA substrate is needed to unambiguously clarify the mechanism of substrate recognition.

The reported relative selectivity of PvuRts1I towards 5-hmC, 5-mC and cytosine is 2000:8:1, indicating that 5-hmC is the preferred substrate. After glucosylation of 5-hmC, the selectivity is even higher (Borgaro & Zhu, 2013 ▶; Szwagierczak *et al.*, 2011 ▶; Wang *et al.*, 2011 ▶). The larger size of 5-hmC may be better accommodated by the wider binding pocket of PvuRts1I, with its fixed shape and volume. The rigidity of the binding pocket may also confer the capacity for discrimination of both 5-hmC and 5-gmC from 5-mC. Consequently, endonucleases of the PvuRts1I family are proposed to be ideal tools for 5-hmC sequencing applications (Sun *et al.*, 2013 ▶; Borgaro & Zhu, 2013 ▶; Szwagierczak *et al.*, 2011 ▶; Wang *et al.*, 2011 ▶).

In a recent report, AbaSI was used to map the genomic distribution of 5-hmC (Sun *et al.*, 2013 ▶). AbaSI only efficiently cleaves substrate DNA containing two 5-hmCs that are 21 or 22 nt apart and on opposite strands. The activity of AbaSI is greatly decreased when only one of the two 5-hmCs is changed to 5-mC or cytosine (Borgaro & Zhu, 2013 ▶; Wang *et al.*, 2011 ▶). However, cytosine and 5-mC are much more abundant in the genome than is 5-hmC, and it is very important to maintain activity when only a single 5-hmC is present on the substrates. A characteristic feature of the activity of PvuRts1I is the similar activity that is displayed on substrates containing either two 5-hmC modifications or one 5-hmC together with one 5-mC or cytosine on the opposite DNA strands (Borgaro & Zhu, 2013 ▶). We therefore suggest that PvuRts1I is suitable for use in this method and may perform better than AbaSI, especially if engineered for higher substrate selectivity. To this end, we mutated PvuRts1I based on structural analysis. Encouragingly, the substrate selectivity of the N217D and particularly the Y210F variant was improved significantly (Figs. 5 ▶ *b* and 6 ▶ *b*). Why Tyr210 plays such a key role in the recognition of 5-hmC remains unknown at present. A crystal structure of PvuRts1I bound to its 5-hmC DNA substrate would be highly informative in order to clarify the substrate-selectivity determinants.

In summary, the crystal structure of PvuRts1I determined in this study, coupled with structural and biochemical analysis, allowed us to engineer enzyme variants that may perform better than AbaSI in the Aba-seq method and may assist in hydroxymethylome mapping and future 5-hmC research.

## Supplementary Material

PDB reference: PvuRts1I, 4oky

Supporting Information.. DOI: 10.1107/S139900471401606X/qh5007sup1.pdf

## Figures and Tables

**Table 1:** Data-collection and refinement statistics Values in parentheses are for the highest shell.

Data collection	
Space group	*P*4_3_2_1_2
Wavelength ()	0.9791
Unit-cell parameters (, )	*a* = *b* = 160.21, *c* = 45.12, = = = 90
Resolution ()	50.02.9 (3.062.90)
Mosaicity ()	0.65
Overall *B* factor from Wilson plot (^2^)	70.6
*R*_merge_ † (%)	12.0 (42.2)
*I*/(*I*)	12.9 (4.6)
Completeness (%)	98.1 (98.0)
Multiplicity	10.5 (10.7)
Unique reflections	13311 (1896)
Refinement	
Resolution ()	50.02.9
*R* factor‡/*R*_free_ § (%)	27.37/29.74
R.m.s. deviations¶	
Bond lengths ()	0.0127
Bond angles ()	1.853
No. of protein atoms	2162
*B* factor (^2^)	55.25
Ramachandran plot††	
Most favoured regions (%)	96.0
Additionally allowed regions (%)	4.0
Outliers (%)	0

† *R*_merge_ = 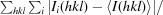 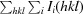, where *I_i_*(*hkl*) is the intensity of the *i*th measurement and *I*(*hkl*) is the mean intensity for that reflection.

‡ *R* factor = 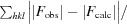 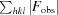, where |*F*_obs_| and |*F*_calc_| are the observed and calculated structure-factor amplitudes, respectively.

§ *R*_free_ was calculated with 5.0% of the reflections in the test set.

¶ R.m.s.d. from ideal values.

†† Categories were defined by *PROCHECK*.

## Footnotes

1 Supporting information has been deposited in the IUCr electronic archive (Reference: QH5007).
