# Supplementary material for: Structural basis for the substrate selectivity of PvuRts1I, a 5-hydroxymethylcytosine DNA restriction endonuclease
Source: Acta Crystallogr D Biol Crystallogr. 2014 Aug 29;70(Pt 9):2477–86. doi: 10.1107/S139900471401606X (PMC4157451; doi:10.1107/S139900471401606X)
Supplement: Supplementary file 1 [file d-70-02477-sup1.pdf]

Supplementary Figures:

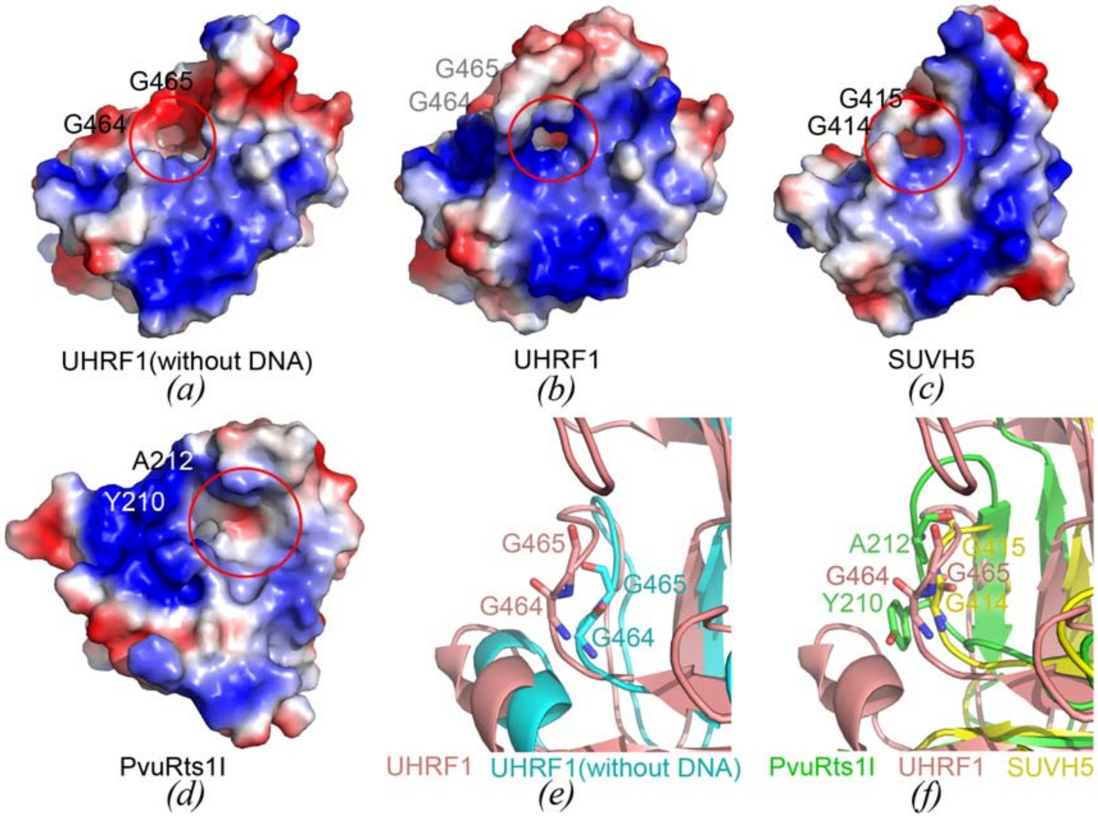

Figure S1. The putative rigid 5-hmC binding site of PvuRts1I. Surface representation of the 5-mC binding pocket of (a) human UHRF1 without substrate DNA (PDB ID 2ZKG), (b) human UHRF1 in complex with substrate DNA (PDB ID 3CLZ), (c) *Arabidopsis* SUVH5 (PDB ID 3Q0F) in complex with substrate DNA. (d) Surface representation of the putative 5-hmC binding pocket of PvuRts1I. Binding pockets are highlighted by red circles in (a)-(d). (e) Comparison of the 5-mC binding pocket of human UHRF1 in the presence or absence of substrate DNA. The loop forming the edge of the binding pocket undergoes a large conformational change. (f) Superimposition of the loops of PvuRts1I, human UHRF1 and *Arabidopsis* SUVH5 that form the edge of binding pocket, in the presence of bound modified cytosine. Amino acid residues are shown in stick mode.

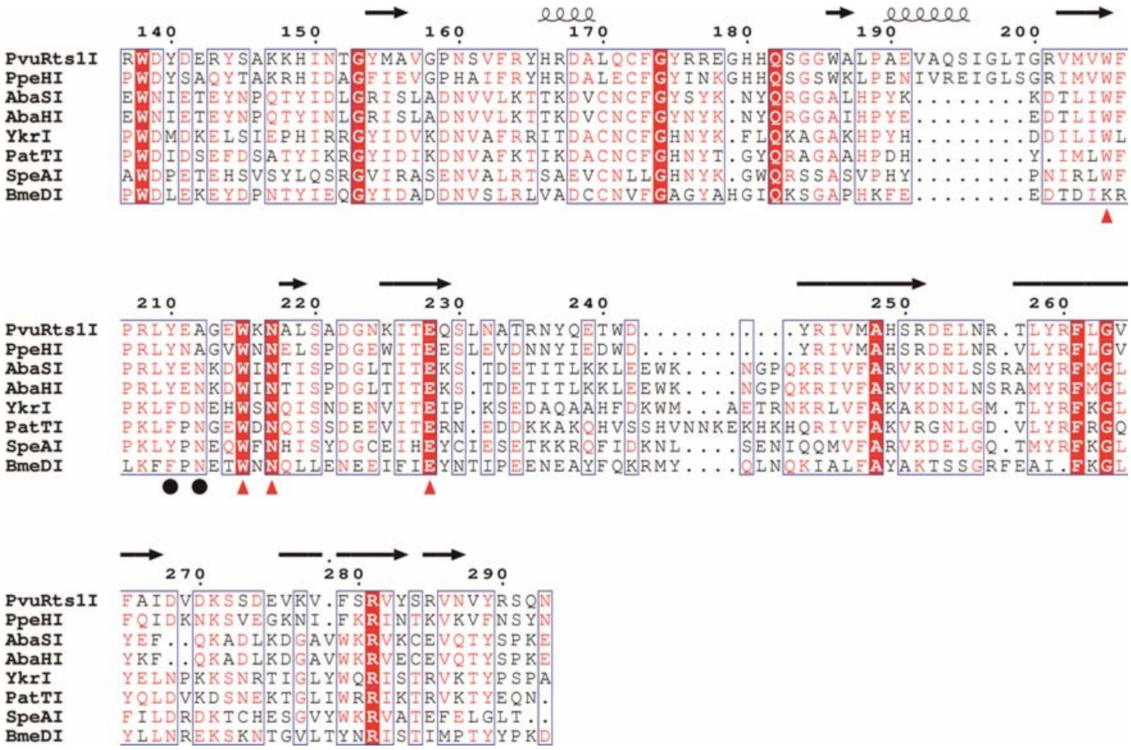

Figure S2. Structure-based sequence alignment of the SRA-like domain of PvuRts1I homologues. Residues potentially involved in the interaction with 5-hmC are marked by a red triangle. Residues proposed to affect the conformation of the 5-hmC binding pocket are marked by black dots.

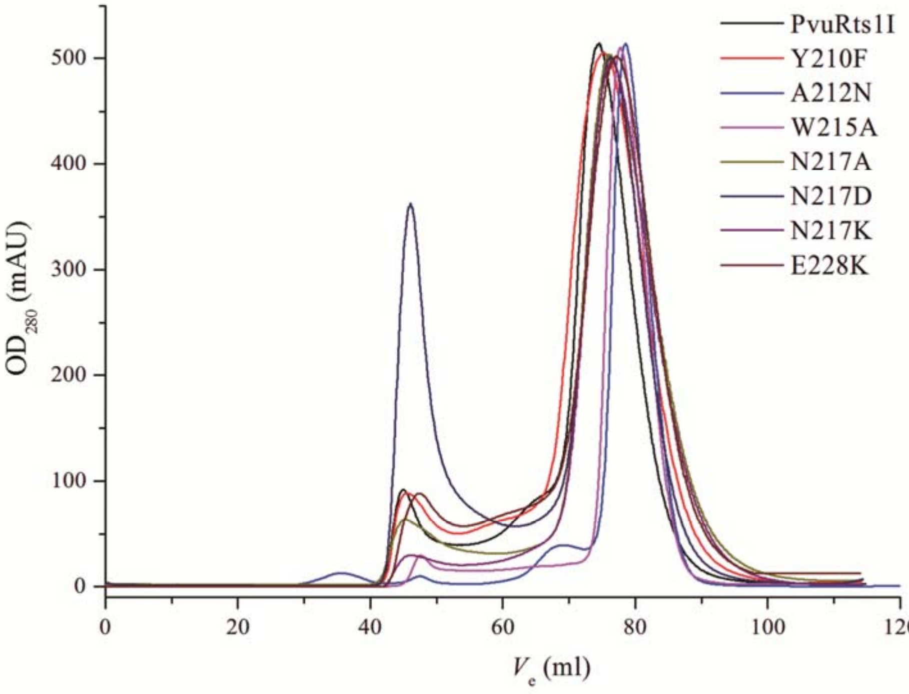

Figure S3. Size-exclusion chromatography analysis of PvuRts1I and its enzyme variants.

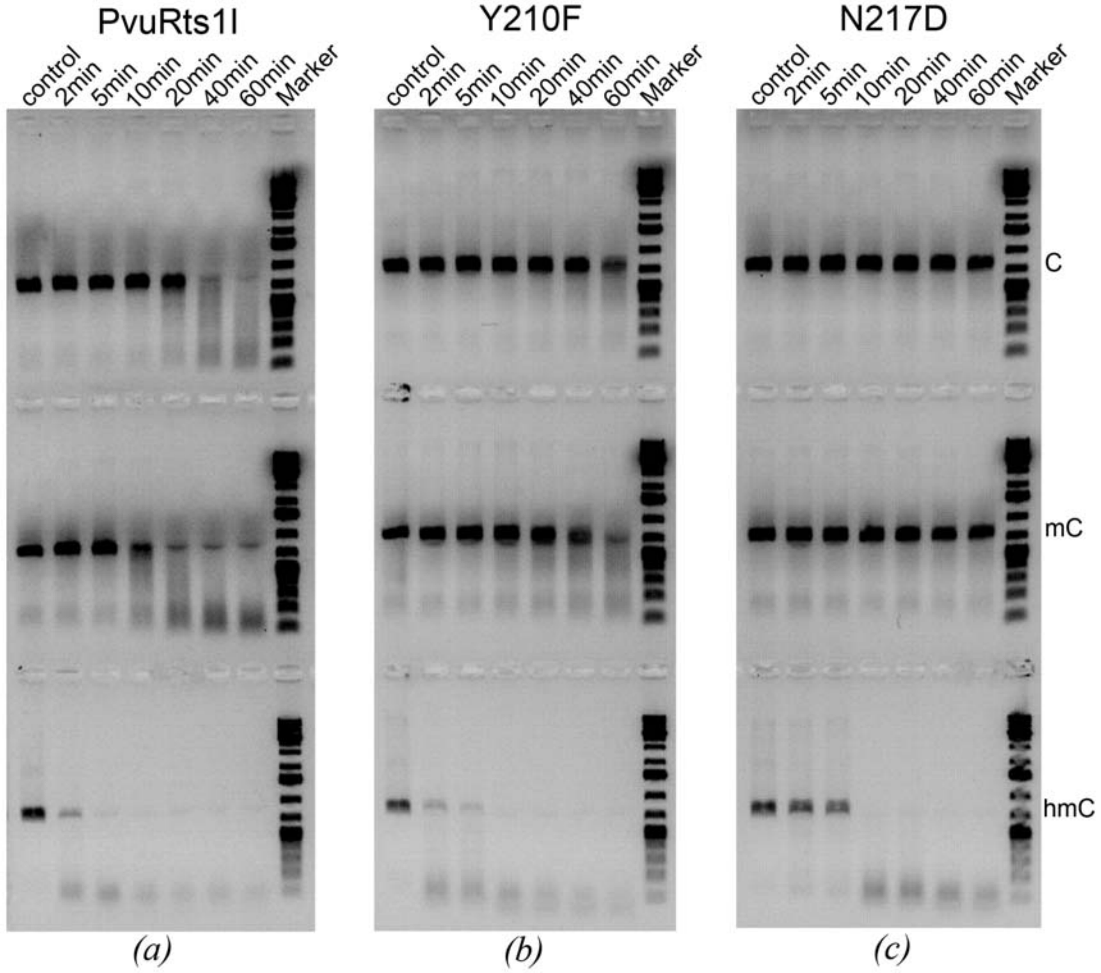

Figure S4. Time-course of the reactions of PvuRts1I and its enzyme variants. (a) PvuRts1I. (b) Y210F. (c) N217D.
